# Shining the light on abortion: Drivers of online abortion searches across the United States in 2018

**DOI:** 10.1371/journal.pone.0231672

**Published:** 2020-05-21

**Authors:** Sylvia Guendelman, Elena Yon, Elizabeth Pleasants, Alan Hubbard, Ndola Prata

**Affiliations:** 1 School of Public Health, University of California, Berkeley (UCB), CA, United States of America; 2 Computer Science Department at UCB, University of California, Berkeley, CA, United States of America; 3 Maternal, Child and Adolescent Health Program, School of Public Health at UCB, University of California, Berkeley, CA, United States of America; 4 Division of Biostatistics at UCB, University of California, Berkeley, CA, United States of America; 5 Bixby Center for Population Health and Sustainability, University of California, Berkeley, CA, United States of America; Emory University School of Public Health, UNITED STATES

## Abstract

**Context:**

Legal abortion restrictions, stigma and fear can inhibit people’s voices in clinical and social settings posing barriers to decision-making and abortion care. The internet allows individuals to make informed decisions privately. We explored what state-level policy dimensions were associated with volume of Google searches on abortion and on the abortion pill in 2018.

**Methods:**

We used Google Trends to quantify the relative search volume (RSV) for “abortion” and “abortion pill” (or “abortion pills” hereafter referred to as “abortion pill”) as a proportion of total search volume for all queries in each US state. We also identified the top search queries most related to “abortion” and “abortion pill” and considered these as indicators of population concern. Key exposures were healthcare cost, access and health outcomes, and number of legal restrictions and protections at the state level. In descriptive analyses, we first grouped the states into tertiles according to their RSV on “abortion” and “abortion pill”. To examine the association between each exposure (and other covariates) with the two outcomes, we used unadjusted and adjusted linear regression.

**Results:**

The average RSV for “abortion” in the low, moderate and high tertile groups was 48 (SD = 3.25), 55.5 (SD = 2.11) and 64 (SD = 4.72) (p-value <0.01) respectively; for “abortion pill” the average RSVs were 39.6 (SD = 16.68), 61.9 (SD = 5.82) and 81.7 (SD = 6.67) (p-value < 0.01) respectively. Concerns about contraceptive availability and access, and unplanned pregnancies independently predicted the relative search volumes for abortion and abortion pill. According to our baseline models, states with low contraceptive access had far higher abortion searches. Volume of abortion pill searches was additionally positively associated with poor health outcomes, poor access to abortion facilities and non-rurality.

**Conclusion:**

Search traffic analysis can help discern abortion-policy influences on population concerns and require close monitoring. State-policies can predict search volume for abortion and abortion pill. In 2018, concerns about contraceptives and unplanned pregnancies, predicted abortion searches. Current decreases in public contraceptive funding and the Title X Gag rule designed to block millions of people from getting care at Planned Parenthood, the largest provider of birth control and abortion care, may increase concerns about unintended pregnancies that can lead to increases in online relative volume of abortion searches.

## Introduction

Abortion rates in the US at 13.5 per 1,000 women ages 15–44 were at an all-time low in 2017, representing a 22% decrease since 2005. Despite the decline, close to 900,000 abortions are performed annually in clinical settings, indicating that it is a very common procedure. [1] Abortion remains a highly contentious and polarizing issue in the US even as abortion and fertility rates fall. Pro-choice experts attribute the drop in abortion to changes in contraceptive use, including greater reliance on highly effective methods, [2–3] and lower rates of unplanned pregnancies [4–5], including teenage pregnancies. [6] Anti-choice groups claim otherwise. Paramount reasons for the decline according to them are a “culture of life” which pushes women towards pregnancy and childbearing [7] and a reduction in facilities at which women can obtain care. [8] Anti-choice groups also link other incremental restrictive policies to the decline [9], even though rates began falling before implementation of a massive number of restrictive laws during 2011–12 and in places with few abortion restrictions. [10,7]

Anti-choice groups strongly believe that abortion should be illegal in most or all cases and have consistently raised barriers in many states, making abortion almost inaccessible. As a result, in 2018, 29 million women of reproductive age in the US lived in states considered hostile or very hostile to abortion. [11] In these states for instance, restrictive policies require mandatory counseling and waiting periods for women seeking an abortion, limiting access to abortion for minors without parental involvement, imposing cumbersome regulations on abortion facilities, and requiring that only a licensed physician perform abortions. [11–13] In opposition, pro-choice groups have recently increased efforts to expand access to abortion and contraception and to protect women’s reproductive rights by enacting laws guaranteeing the legality of abortion if *Roe v*. *Wade* were to be overturned, requiring insurance coverage of abortion services, [12] and by repealing legislation that creates barriers to accessing abortion. [13] Access has also been expanded with the use of telemedicine to administer medication abortion, and by FDA approval for use of medication abortion until later in pregnancy, allowance of administration by non-physicians, and lowered costs. [14]

Increased legal restrictions, stigma around abortion, and fear of possible legal consequences if a woman were to intentionally terminate her pregnancy, pose barriers to information seeking in clinical settings. [15] Stigma and lack of access to information have been linked to the use of the internet as a resource for abortion information and services. [16–17, 8] By allowing information seeking to be done privately or by connecting people to other resources, the internet gives individuals the chance to make more informed, autonomous decisions. [18] Yet, navigating these resources on the internet can be difficult especially for individuals at high risk for poor health consequences who may face barriers related to misinformation and biased search results from anti-choice websites. [19]

According to two previous studies using Google search data, people in more restrictive states are more prone to engage in online abortion searches. [20–21] One reason for the heightened search activity might be that individuals do not know where to access care as more clinics close or do not facilitate referrals. [8, 19] A study of Google search queries across US states found that populations in states with a high search index for abortion had higher teenage pregnancy rates than those in states with a lower search index. [22] In contrast, states with a high search index for condoms had a low teenage birth rate. [22] Presumably, greater access to health care in general seems to facilitate the abortion care and contraceptive care that prevents unintended pregnancy. [1] However, the extent to which rates of unintended pregnancies or the performance of the health system including costs, access and outcomes of care, influence abortion search traffic is not known. These factors along with abortion restrictions and protections show great variability across states and may influence the extent to which people go online to search for abortion information and services or to reduce their level of perceived risk for undesirable outcomes. It is possible that in states with cost, geographical access, and informational barriers to reproductive care, and to health care barriers in general, internet seeking on abortion and contraceptive care is higher than in states where health systems perform better. On the other hand, the increased availability of abortion pills through online sources and a preference for self-management to bypass restrictive abortion care or poor quality care, may spur women to search online for information on self-abortion. [17]

The purpose of this paper was to explore what key policy-related factors at the state level are currently associated with the volume of online abortion searches, and more specifically, with abortion pill searches across the US. We built separate models to identify the extent to which legal restrictions and protections, availability of abortion facilities, health care costs, access and health outcomes, opinions about abortion, concern about birth control, and prevalence of unplanned pregnancies drive the volume of online searches for abortion and for the abortion pill. We focus on the abortion pill since prior research has shown that the abortion pill is the top abortion procedure searched for on Google in the nation. [17, 23] Since the majority of people search for health information using Google [24] our analysis was restricted to this search engine.

## Methods

### Sample and measures

This cross-sectional, ecological study used Google Trends, a publicly available online tool which allows download of web-search data for specific keywords. Using the “Explore” feature, we compared online interest on two broad search terms, “abortion” and “abortion pill” in each of the 50 states. We searched within the US from January 1 to December 31, 2018 using the “health” query category. We chose January 1, 2018 as the start date to capture baseline interest in the year for which most complete data were available. We used the Google Trends proprietary API (Applied Program Interface) to request with “getTopQueries” a list of search queries that were most related to the two broad search terms. The top search queries for “abortion” were abortion pill, abortion pill cost, abortion clinic(s), Planned Parenthood, abortion facts, abortion statistics and partial birth abortion. Top queries for “abortion pill” were abortion pill cost, Planned Parenthood, morning after pill, plan b, plan b pill, take action pill, abortion clinics, abortion pill online and free abortion clinics. These search queries were considered indicators of population concern and unmet needs.

We obtained the relative search volume (RSV) i.e. the volume of searches for the two broad terms as a proportion of the total search volume for all queries across a given state. The RSV is a normalized score provided by Google Trends that represents the relative popularity of “abortion” and “abortion pill” as searched terms in a given state. Scores range from 0 (if search volume is low (or below the threshold of search traffic acceptable to Google for privacy protection) to 100 (highest) for a given representative sample of searches in the population. [25]

Although this normalized score accounts for the total search volume in a region in a given period and makes data from different states comparable with each other, the exact volume of queries for each state are not reported. Google automatically removes repeated queries over a short time-period from a single user to control for artificial effects. [25]

Our key exposures of interest consisted of an array of health systems dimensions and number of legal restrictions and protections that set the context of care at the state level, drawn from separate sources. *Health systems variables* consisted of the cost of care, access to care, health outcomes, and overall health system’s performance rankings defined as the best care for the most reasonable cost in each state in 2017, drawn from Wallethub (wallethub.com/edu/states-with-best-health-care/23457/). Wallethub is a personal financial website that uses 40 metrics to annually evaluate each state’s *cost of care* (e.g.: cost of medical visit; cost of dental visit; average insurance premiums; share of out of pocket spending); *access to care* (e.g.: quality of public hospital system; average emergency wait time and visit time; per capita hospital beds, number of physicians, nurse practitioners, and urgent care centers; share of insured children; presence of telehealth); and *health outcomes* (e.g.: rates of infant mortality, maternal mortality, cancer, heart disease, type 2 diabetes, life expectancy). State rankings range from best (score = 1) to worst (score ≤ 100) on each of these dimensions with each dimension receiving a maximum score of 33.3 points, adding up to 100 points. The overall health system’s performance score is the weighted average score across the three dimensions.

*Legal context* refers to the number of legal restrictive measures against abortion and the number of legal abortion protections implemented in each state. In 2018, the Guttmacher Institute [12] and/or NARAL Pro-Choice America [26] identified 28 potential restrictions (Table 1). The observed range across states was 0–23. The *number of legal protections*, which according to NARAL a state could have to allow or guarantee abortions, comprised up to 11 potential measures in 2018 (Table 1). The observed range was 0–8.

**Table 1:** Indicators of legal context for abortion: Restrictions and protections.

**Restrictions**
1. abortions must be performed by a licensed physician;
2. abortions must be performed at a hospital starting at a time-point in gestation;
3. abortions must be performed by providers with hospital admitting privileges;
4. second physician must participate after a certain point in gestation;
5. some or all state employees or organizations that receive state funds are prohibited from providing, counseling or referring women for abortion services;
6. abortions are prohibited except when necessary to protect the woman’s life or health;
7. abortion coverage in private insurance plans is restricted, mostly to when the woman’s life would be endangered if the pregnancy were to be carried to term, or the pregnancy is the result of rape, incest or when federal funds are available;
8. public insurance coverage is limited to situations of life endangerment, rape, incest or when federal funds are available;
9. explicitly stipulates that individual health care providers may refuse to participate in an abortion;
10. health care institutions can refuse to participate in the provision of abortion;
11. State mandates that women be given counseling before an abortion that includes information on:
12. the purported link between abortion and breast cancer
13. the ability of a fetuses to feel pain
14. the potential for medication abortions to be reversed following taking the first pill in the regimen
15. the long-term mental health consequences for the woman;
16. requires a woman seeking an abortion to wait a specified time period, usually 24 hours, between when she receives counseling and the procedure is performed;
17. requires a woman to receive an ultrasound prior to the abortion;
18. requires parental involvement (notification and/or consent) in a minor’s decision to have an abortion;
19. has a law specifically banning “partial birth abortion”;
20. has a ban on “dilatation and evacuation” procedure;
21. has near-total criminal bans on abortion;
22. requires that medication abortion be provided by a licensed physician;
23. requires that a physician be physically present for a medication abortion, outlawing telemedicine provision of abortion;
24. has a law that would require near-total criminal bans on abortion if the Supreme Court overturns Roe vs. Wade;
25. supports Crisis Pregnancy Centers by funding these centers or mandating referrals to these centers;
26. bans research involving fetal tissue research;
27. requires that fetal tissue be cremated or buried;
28. prohibits schools from discussion of abortion or referring students to abortion services.
**Protections**
**1.** Has a measure guaranteeing public insurance coverage for abortion services;
2. state funds abortion services for low-income women beyond life endangerment, rape and/or incest or imposes no restrictions on low-income women’s abortion services;
3. state constitution provides greater protection than the federal constitution of a woman’s right to choose;
4. has enacted legislation to improve protections for abortion access in state law;
5. has codified a woman’s right to choose, making the protections of Roe vs. Wade part of state law;
6. has measures that protect healthcare facilities, providers and/or patients from blockades, harassment and/or other anti-choice violence;
7. has measures opposing Crisis Pregnancy Centers to ensure that women have accurate information about the full range of reproductive-health services;
8. has laws that improve access to emergency contraceptives;
9. has expanded the scope of practice of advanced-practice clinicians to include medication and/or surgical abortions;
10. allows other qualified healthcare professionals to provide surgical abortion;
11. requires that abortion be discussed in sex education courses as a positive option to manage pregnancy.

Sources: Guttmacher Institute, 2018^12^ and NARAL Pro-Choice America, 2018^24^

Other independent variables included for each state were measures previously shown to contribute or hinder access to abortion care. These were: the number of abortion facilities performing abortions; [27] the number of women ages 15 to 49 years per abortion facility obtained from a systematic search of online abortion facilities in each state conducted in early 2017 by Cartwright and co-investigators; [8] the percent of adults who thought that abortion should be illegal in all or most cases in 2014 as reported by a Pew Research Center survey; [28] the percent of unintended pregnancy in 2010—the latest rates available at the time of analysis; [29] and the relative volume of online searches for birth control which is an indicator of contraceptive methods and attributes that people search for and which we generated from the Google Trends website. In addition, two demographic variables obtained from the US Census were included as covariates, the percent of a state’s population living in rural areas [30] and the proportion of the population aged 18–24 years out of the total population in a given state in 2017. [31]

### Analysis plan

We first grouped the 50 states into tertiles according to their RSV for “abortion” and their RSV for “abortion pill”. States in the lowest tertile had low RSV, those in the second tertile had moderate RSV, and those in the highest tertile had high RSV. We initially excluded the District of Columbia (DC) from the analysis because it is the hub of politics, and searches on issues such as abortion might become exacerbated. In fact, the RSV for abortion indicated that it was an outlier, with much higher search volume than all other states. For each of the three groups of states, we calculated means and standard deviations (SD) for each of the key exposures, namely health care systems scores and the number of legal restrictions and protections, as well as for the other covariates of interest. This allowed us to characterize the profile of policy levers prevalent in each group. We performed analysis of variance (ANOVA) F-tests to compare group mean differences, and then followed up the significant ones with t-tests, using the Dunn-Bonferroni multiple comparison procedure, comparing the t-test p values to .05/3 = .0167. [32] We report significant differences at a p value < = 0.017.

Subsequently we standardized variables to make it easier to compare regression coefficients by subtracting the mean of each independent variable from the original value for each state divided by the SD. We examined the association between each standardized predictor and the outcomes ranked-ordered across states using simple linear regressions. Unadjusted beta coefficients estimated the strength of the correlations and p values were used to denote level of significance. Since our unit of analysis was the state (thus only 50 units), we limited the number of potential predictors that could be included in the multivariable models to avoid overfitting. Only those variables that in bivariate analysis were significantly correlated with the outcomes at p< = 0.10 were included as potential predictors. We performed a forward stepwise regression model to select the best set of predictors from this pool. The forward model starts with no variables in the model and stops when no new predictors can be added to the model. We used the Akaike Information Criterion (AIC) to choose the best fitting model with the fewest number of parameters. [33] AIC is a measure of likelihood used to approximate the relative mean-squared error of models. We performed sensitivity analyses with DC in and out of the model to assess predictors. We ran bivariate analyses in Python and multivariable regressions in R.

## Findings

### a. Abortion searches

The three groups of states differed in their search volume for abortion relative to searches for other queries in each state. The RSV ranged from 40 (lowest) in Hawaii to 77 (highest) in Mississippi and the average RSV in the low, moderate, and high tertile groups was 48 (SD = 3.25), 55.5 (SD = 2.11) and 64 (SD = 4.72) (S1 Table). The three groups differed on several health related and legal characteristics. Compared to the moderate tertile group, the high tertile group had on average higher proportions of unintended pregnancy and compared to the low tertile group, the high tertile group had on average health care systems with worse health care performance and worse health outcomes, more legal restrictions for abortion, fewer abortion protections, more people opining that abortion should be illegal in all or most cases, and higher unintended pregnancy (Table 2).

**Table 2:** Health and demographic characteristics of US States with low, moderate, and high Relative Search Volume (RSV) for abortion in 2018*.

	1. Low RSV		2. Moderate RSV		3. High RSV		t-test
	mean	SD	mean	SD	mean	SD	p-value**
**Health Systems Performance Score** ^+^	18.19	13.79	27.46	14.16	31.90	13.66	b
Cost	29.94	12.36	23.08	13.83	26.00	16.55	
Access^+^	21.31	16.74	26.38	12.16	30.29	14.02	
Health outcomes^+^	11.81	7.33	29.15	16.18	34.38	10.43	b, c
**Number abortion restrictions**	7.75	4.61	12.15	6.79	13.10	6.02	b
**Number of abortion protections**	2.88	2.42	1.77	2.01	1.29	1.65	b
**Number of abortion facilities**	18.94	36.78	4.85	3.39	19.57	23.13	
**Number of women ages 15–49 per abortion facility**	101,197	92,505	313,316	404,700	202,094	158,179	
**% Opine that abortion should be illegal**	0.38	0.09	0.45	0.11	0.46	0.08	b
**% Unintended pregnancy**	45.44	4.80	49.69	4.21	54.29	4.29	a, b
**RSV for birth control (2018)**	70.63	3.93	76.62	6.89	81.52	7.97	a, b, c
**% Population 18–24 years old**	0.08	0.01	0.08	0.00	0.08	0.00	
**% Rural population**	25.59	16.44	28.36	15.73	25.24	12.72	

* Low RSV: Hawaii, Utah, Montana, Wyoming, Idaho, Colorado, Washington, New Hampshire, South Dakota, Maine, Connecticut, Massachusetts, California, Nebraska, Alaska, Minnesota

Moderate RSV: New Mexico, Wisconsin, Oregon, Rhode Island, Missouri, Kansas, Arizona, Arkansas, Tennessee, Vermont, Kentucky, Nevada, North Dakota

High RSV: Texas, South Carolina, New Jersey, Florida, Oklahoma, Virginia, Delaware, Pennsylvania, Ohio, New York, Indiana, North Carolina, Illinois, Michigan, Iowa, Maryland, West Virginia, Louisiana, Georgia, Alabama, Mississippi

+ lower score denotes better performance

**t-tests denote significant differences between groups using the Dunn-Bonferroni multiple comparison procedure comparing the p-values to .05/3 = .0167

a: p-value for significant differences in group 3 versus group 2

b: p-value for significant differences in group 3 versus group 1

c: p-value for significant differences in group 2 versus group 1

Compared to the moderate tertile group, the low tertile group had on average better health outcomes and lower searches on birth control (Table 2). The three groups did not differ significantly on access or cost of care, number of abortion facilities, or number of women ages 15–49 per facility, percent of population 18–24 years out of the total state population, or percent living in rural areas.

In unadjusted models, worse overall health care system performance, worse health outcomes, a higher number of women ages 15–49 per abortion facility, a higher number of legal restrictions and of abortion protections, a higher proportion of adults opining that abortion should be illegal, higher unintended pregnancy, higher volume of birth control searches, and a young population 18–24 were independently and significantly correlated with higher abortion RSV (Table 3). After adjusting for all the variables significant in unadjusted models, only volume of birth control searches and unintended pregnancy remained significant positive predictors of abortion searches (Table 3).

**Table 3:** Unadjusted and adjusted regression coefficients for Relative Search Volume (RSV) for “Abortion” in 2018.

Independent Variable	Unadjusted Coefficient (Beta)	p-value	Adjusted Coefficient (Beta)	p-value
**Health Systems Performance Score** ^**+**^	0.39	≤0.001		
Cost	-0.09	0.55		
Access^+^	0.19	0.18		
Health outcomes^+^	0.66	≤0.001		
**Number abortion restrictions**	0.35	0.01		
**Number of abortion protections**	0.33	0.02		
**Number of abortion facilities**	-0.13	0.36		
**Number of women ages 15–49 per abortion facility**	0.32	0.02		
**% Opine that abortion should be illegal**	0.33	0.02		
**% Unintended pregnancy**	0.63	≤0.001	0.39	≤0.001
**RSV for birth control (2018)**	0.70	≤0.001	0.52	≤0.001
**% Population 18–24 years old**	0.28	0.05		
**% Rural population**	0.09	0.51		

+ lower score denotes better performance

### b. Abortion pill searches

The RSV for the abortion pill ranged from 0 (too low to be reported by Google) in Wyoming to 100 in Georgia and the average RSV was 39.6 (SD = 16.68) in the low tertile group, 61.9 (SD = 5.82) in the moderate, and 81.7 (SD = 6.67) in the high tertile group (S1 Table). On average, the high tertile group had significantly higher proportions of unintended pregnancy compared with the moderate tertile group (Table 4). Compared with the low tertile group, the high tertile group had on average lower overall health system’s performance and poorer health outcomes, a higher number of abortion facilities, and higher unintended pregnancy. Compared with the moderate tertile group, the low tertile group had better health outcomes, lower proportions of unintended pregnancy, and lower volume of birth control searches (Table 4).

**Table 4:** Health and demographic characteristics of US States with low, moderate, and high Relative Search Volume (RSV) for abortion pill in 2018*.

	1.Low RSV		2.Moderate RSV		3.High RSV		t-test
	mean	SD	mean	SD	mean	SD	p-value**
**Health Systems Performance Score** ^+^	18.31	13.03	26.88	14.92	33.41	13.05	b
Cost	28.06	14.33	23.82	13.32	27.71	16.41	
Access^+^	21.13	15.04	23.53	12.78	34.24	13.71	
Health outcomes^+^	12.75	7.95	30.18	16.69	33.71	9.45	b, c
**Number abortion restrictions**	8.88	5.69	13.00	6.42	11.41	6.05	
**Number of abortion protections**	2.00	2.13	1.76	1.92	2.00	2.32	
**Number of abortion facilities**	8.44	9.16	7.65	6.92	30.12	40.03	b
**Number of women ages 15–49 per abortion facility**	128,154	122,110	289,826	354,910	174,043	167,166	
**% Opine that abortion should be illegal**	0.41	0.08	0.45	0.11	0.44	0.09	
**% Unintended pregnancy**	44.06	3.40	51.47	3.73	54.88	3.92	a, b, c
**RSV for birth control (2018)**	71.19	3.64	79.29	8.45	79.47	8.20	b, c
**% Population 18–24 years old**	0.08	0.01	0.08	0.00	0.08	0.01	
**% Rural population**	32.40	15.00	26.31	13.41	20.15	13.37	

* Low RSV: Hawaii, Utah, Montana, Wyoming, Idaho, Colorado, Washington, New Hampshire, South Dakota, Maine, Connecticut, Massachusetts, California, Nebraska, Alaska, Minnesota

Moderate RSV: New Mexico, Wisconsin, Oregon, Rhode Island, Missouri, Kansas, Arizona, Arkansas, Tennessee, Vermont, Kentucky, Nevada, North Dakota

High RSV: Texas, South Carolina, New Jersey, Florida, Oklahoma, Virginia, Delaware, Pennsylvania, Ohio, New York, Indiana, North Carolina, Illinois, Michigan, Iowa, Maryland, West Virginia, Louisiana, Georgia, Alabama, Mississippi

+ lower score denotes better performance

**t-tests denote significant differences between groups using the Dunn-Bonferroni multiple comparison procedure comparing the p-values to .05/3 = .0167

a: p-value for significant differences in group 3 versus group 2

b: p-value for significant differences in group 3 versus group 1

c: p-value for significant differences in group 2 versus group 1

In unadjusted regression models, lower overall health system’s performance, worse access to care, worse health outcomes, lower number of abortion facilities, higher unintended pregnancy, higher birth control search volume, and a lower percent of population living in rural areas were independently and significantly correlated with higher abortion pill searches (Table 5). After adjusting for these significant variables, only five remained significant predictors of abortion pill searches, namely unintended pregnancies, health outcomes, number of abortion facilities, rural population, and volume of birth control searches.

**Table 5:** Unadjusted and adjusted regression coefficients for Relative Search Volume (RSV) for “Abortion pill” in 2018.

Independent Variable	Unadjusted Coefficient (Beta)	p-value	Adjusted Coefficient (Beta)	p-value
**Health Systems Performance Score** ^+^	0.41	≤0.001		
Cost	-0.03	0.84		
Access^+^	0.38	0.01		
Health outcomes^+^	0.56	≤0.001	0.34	≤0.001
**Number abortion restrictions**	0.12	0.41		
**Number of abortion protections**	0.02	0.91		
**Number of abortion facilities**	-0.39	≤0.001	-0.30	≤0.001
**Number of women ages 15–49 per abortion facility**	0.08	0.58		
**% Opine that abortion should be illegal**	0.01	0.95		
**% Unintended pregnancy**	0.78	≤0.001	0.36	≤0.001
**RSV for birth control (2018)**	0.44	≤0.001	0.20	0.05
**% Population 18–24 years old**	0.20	0.17		
**% Rural population**	-0.37	0.01	-0.29	≤0.001

+ lower score denotes better performance

## Discussion

There are wide variations in the policy decisions that states make regarding abortion and abortion care and these decisions are in flux. This exploratory study examined potential state motivators of online search traffic for abortion and the abortion pill relative to overall search volume in US states in 2018 and presents hypothesized relationships between influential state contextual factors and searches. Interest in abortion looms large. In 2017, according to one study approximately 16.5 million to over 18 million searches for abortion took place nationwide on Google. [23] Compare this with diabetes, one of the leading chronic diseases in the US; there were almost 48 million searches for “diabetes during a three year period (2015–2017). [34]

Our findings show that after adjusting for other factors, the RSVs for both abortion and abortion pill are independently associated with two predictors, volume of birth control searches and the percent of pregnancies that were unintended. States with higher unintended pregnancy rates and states with higher volume of birth control searches—which tend to be states that do not support access to contraception such as Alabama, Louisiana, and Mississippi [35–37]—have significantly higher search traffic on abortion, suggesting a high level of unmet need for information on contraceptives and perhaps for reproductive services overall.

Concerns about method efficacy, cost, availability, and location of care lead people to search for birth control information online. [23] Although we cannot rule out reverse causality, it is possible that searches for birth control drive searches for abortion as women weigh options with an awareness that they have been or could be unsuccessful in preventing unintended pregnancy. The dissemination of misinformation in popular media and politics, including the conflation of certain birth control methods with abortifacients, [38] could add to confusion around contraceptive and abortion methods and potentially lead to more searches for both birth control and abortion options. Furthermore, as prior research on women who discover an unwanted or unintended pregnancy shows, the decision to keep or terminate a pregnancy requires an appraisal process prior to making a decision. [16] It is plausible that this appraisal process leads to online searches for information about abortion, including the abortion pill, and for self-management of medication abortion. [17]

As for broader dimensions of the health system, only health outcomes were important predictors of abortion searches after controlling for other factors. Worse health outcomes in a state were associated with more searches for the abortion pill and for abortion when DC was included in the model. States with worse health outcomes have disproportionately higher rates of maternal, infant and child mortality, and chronic diseases such as obesity and hypertension. [39–40] Previous research has also shown that states funding abortion for low-income women and spending more on contraception have improved maternal and child health outcomes. [41–42] In contrast, states with more restrictive access to abortion tend to implement fewer policies known to support women and children’s health. [43] Our study attempts to make a connection between state-level health outcomes and volume of abortion pill searches, and potentially also about abortion searches. The relationship between health outcomes, healthcare quality, and abortion seeking behaviors needs further exploration to understand how health and healthcare constraints shape experiences for those seeking online information on abortion or services.

Compared with searches for abortion, searches for the abortion pill appear to be more sensitive to access barriers, specifically to access to abortion facilities. We found that states with fewer abortion facilities had higher RSV for the abortion pill after controlling for other factors. Recent evidence shows that closures of abortion facilities and barriers to access increase abortion searches online. [8,18] In recent years, the targeted regulation of abortion providers has led to the shutdown of many clinics and forced women to travel long distances to access services. In 2017, 27 US cities were considered “abortion deserts” because their nearest clinic was 100 miles away. [8] Women living in rural areas are more likely to travel farther for services and face additional barriers in access related to the absence of public transportation that requires money and time resources to access an abortion clinic. [8] The compounding challenges of lack of facilities, highly burdensome restrictions that reduce access to the few clinics, stigma attached to fertility control, and lower access to internet in rural areas may discourage women from seeking information online. [16, 7]

Notably, just as restrictive state laws are likely not driving the decrease in abortion rates, [7] we found that the number of restrictive state laws do not directly predict searches for the abortion pill. This finding is consistent with recent evidence showing that restrictive laws do not necessarily deter individuals seeking medication abortion online. [17] Inquiries into the use of, and of, or ordering medication abortion pills can be done online, thereby bypassing clinic or provider restrictions. [44, 18]

As for abortion searches, we found that the number of legal restrictions was correlated with a larger search volume only in unadjusted, not adjusted models. Over 400 abortion restrictions were enacted in the US since 2010, and while some were challenged in court, most restrictions remain under effect. [9] Future research should analyze whether restrictions that are more coercive, particularly those intended to make abortion too expensive or burdensome for low-income women, have a stronger effect on abortion search volume.

Similarly, rival policy protections that support and expand family planning choices for women, including abortion care, are also correlated with a higher volume of searches for abortion in unadjusted, not adjusted analyses. Furthermore, legal protections do not correlate with searches for the abortion pill, perhaps because in contexts where abortion access is limited, a substantial number of people may opt to bypass the healthcare systems and perform medication abortion at home. In contexts that support abortion access, people are more likely to seek information and access to the abortion pill in-person through healthcare providers. While currently the internet is a key source of information on abortion, evidence suggests that women generally prefer to seek abortion information from healthcare providers [45] but that young, college-age women most often turn first to the internet for information related to sexual health concerns followed by healthcare providers. [46]

Search engine data are underutilized real-time data sources for studying the impact of public health policies on population interests and information needs that contribute to decision making on health. As a next step, our methodology could be useful in identifying whether the same or different state policy levers contribute to online interests and concern about abortion and the abortion pill beyond 2018. Stratified analyses could determine whether the policy-related factors differ in rural and urban states and in states with large populations of color. Future studies should also assess the policy-related factors that contribute to online searches for contraceptives over time and across states, since contraceptive queries are important predictors of abortion searches. The findings from these studies can complement survey and clinic data to guide health systems and program planners in their effort to support reliable and actionable sexual and reproductive health information, health resources and education online.

While this study presents novel and timely data on policy levers of Google search traffic on abortion and the abortion pill, it has limitations. This cross-sectional study does not allow us to infer causality or the direction of the relationship between search volume on abortion or abortion pill and the examined predictors. The ecological nature of the study using aggregated and ranked data for each state does not allow us to ascertain the characteristics of the individuals searching for the information or the reasons for their search. We used top queries associated with the key broad search terms as proxies for population concerns and unmet needs. Previous studies have shown varying health search use across demographic and geographic populations and more online health searches performed by women than men. [22] We restricted the study to searches in English. Additionally, the internet is still inaccessible for some in the US, with broadband coverage rates as low as 70% in some states in 2018 (Montana and Mississippi). [47] Moreover, in conservative states, abortion stigma is so high that some people might be unwilling to initiate a search for fear of potential repercussions. There is need for further research to explore who are the disenfranchised groups unable to use the internet as a resource for information on abortion as well as explore how use of the internet may interact and intersect with other sources of information (social networks, healthcare providers, etc.). Our models assessed leading policy issues at the state-level to capture the socio-political and healthcare context using most up-to-date information. However, data on unintended pregnancies were from 2010 since more recent state-level data were not available. We did not account for the possibility that media attention to abortion can influence online abortion searches. A recent communication study of Google searches demonstrated low correlation of mainstream media or new media coverage with abortion search queries. [48]

In conclusion, there is a compelling need to understand and monitor internet searches in our rapidly changing legal and health care context where personal decisions are increasingly under state control. Our findings indicate that certain state policy-related factors predict search volume of abortion and abortion pill. In 2018, concerns about availability along with access to and costs of contraceptives and unplanned pregnancies (which are directly related to contraceptive use), not the incremental number of restrictive state abortion laws, predicted abortion search traffic. Increased search volume of abortion pill was additionally driven by poor state-level health outcomes, poor access to abortion facilities, and non-urban locations. Current government cuts backs of contraceptive availability and funding, denial of accurate information and, the Title X Gag rule designed to block millions of people from getting care at Planned Parenthood, the largest provider of birth control and abortion care, may increase concerns about unintended pregnancies that can lead to increases in online relative volume of abortion searches [49].

## Supporting information

S1 Table Relative search volume (RSV) for “abortion” and “abortion pill” in the US for 2018. (DOCX)
