# Supplementary material for: Shining the light on abortion: Drivers of online abortion searches across the United States in 2018
Source: PLoS One. 2020 May 21;15(5):e0231672. doi: 10.1371/journal.pone.0231672 (PMC7241764; doi:10.1371/journal.pone.0231672)
Supplement: S1 Table — (DOCX) [file pone.0231672.s001.docx]

**SUPPLEMENTARY INFORMATION**

**S1 Table. Relative Search Volume (RSV) for “abortion” and “abortion pill” in the US for 2018**

Source: Google Trends Website, accessed on 4/29/2019

| **State** | **RSV for "abortion"** | **median** | **mean** | **SD** | **p-val** |  | **State** | **RSV for "abortion pill"** | **median** | **mean** | **SD** | **p-val** |
| --- | --- | --- | --- | --- | --- | --- | --- | --- | --- | --- | --- | --- |
| Hawaii | 40 | 49.00 | 48.00 | 3.25 | <0.01 |  | Vermont | 0 | 44.00 | 39.63 | 16.68 | <0.01 |
| Utah | 43 |  |  |  |  |  | Wyoming | 0 |  |  |  |  |
| Montana | 45 |  |  |  |  |  | North Dakota | 32 |  |  |  |  |
| Idaho | 46 |  |  |  |  |  | South Dakota | 37 |  |  |  |  |
| Wyoming | 46 |  |  |  |  |  | Utah | 39 |  |  |  |  |
| Colorado | 47 |  |  |  |  |  | Nebraska | 40 |  |  |  |  |
| New Hampshire | 49 |  |  |  |  |  | Idaho | 41 |  |  |  |  |
| South Dakota | 49 |  |  |  |  |  | Maine | 42 |  |  |  |  |
| Washington | 49 |  |  |  |  |  | Minnesota | 46 |  |  |  |  |
| Connecticut | 50 |  |  |  |  |  | Oregon | 48 |  |  |  |  |
| Maine | 50 |  |  |  |  |  | Wisconsin | 50 |  |  |  |  |
| Massachusetts | 50 |  |  |  |  |  | Washington | 51 |  |  |  |  |
| Alaska | 51 |  |  |  |  |  | Alaska | 52 |  |  |  |  |
| California | 51 |  |  |  |  |  | Colorado | 52 |  |  |  |  |
| Minnesota | 51 |  |  |  |  |  | Iowa | 52 |  |  |  |  |
| Nebraska | 51 |  |  |  |  |  | New Hampshire | 52 |  |  |  |  |
| New Mexico | 53 | 55.00 | 55.46 | 2.11 | <0.01 |  | Hawaii | 53 | 62.00 | 61.88 | 5.82 | <0.01 |
| Oregon | 53 |  |  |  |  |  | Massachusetts | 54 |  |  |  |  |
| Wisconsin | 53 |  |  |  |  |  | Montana | 54 |  |  |  |  |
| Kansas | 54 |  |  |  |  |  | Kansas | 56 |  |  |  |  |
| Missouri | 54 |  |  |  |  |  | Missouri | 57 |  |  |  |  |
| Rhode Island | 54 |  |  |  |  |  | Kentucky | 59 |  |  |  |  |
| Arizona | 55 |  |  |  |  |  | Arkansas | 60 |  |  |  |  |
| Arkansas | 56 |  |  |  |  |  | West Virginia | 61 |  |  |  |  |
| Tennessee | 57 |  |  |  |  |  | Tennessee | 62 |  |  |  |  |
| Kentucky | 58 |  |  |  |  |  | Connecticut | 64 |  |  |  |  |
| Nevada | 58 |  |  |  |  |  | New Mexico | 64 |  |  |  |  |
| North Dakota | 58 |  |  |  |  |  | Ohio | 65 |  |  |  |  |
| Vermont | 58 |  |  |  |  |  | Oklahoma | 66 |  |  |  |  |
| Florida | 59 | 63.00 | 64.00 | 4.72 | <0.01 |  | Virginia | 67 |  |  |  |  |
| New Jersey | 59 |  |  |  |  |  | Indiana | 68 |  |  |  |  |
| Oklahoma | 59 |  |  |  |  |  | Rhode Island | 70 |  |  |  |  |
| South Carolina | 59 |  |  |  |  |  | Texas | 72 |  |  |  |  |
| Texas | 59 |  |  |  |  |  | Arizona | 74 | 80.00 | 81.65 | 6.67 | <0.01 |
| Virginia | 61 |  |  |  |  |  | Louisiana | 74 |  |  |  |  |
| Delaware | 62 |  |  |  |  |  | Pennsylvania | 75 |  |  |  |  |
| New York | 62 |  |  |  |  |  | California | 77 |  |  |  |  |
| Ohio | 62 |  |  |  |  |  | Delaware | 77 |  |  |  |  |
| Pennsylvania | 62 |  |  |  |  |  | Michigan | 78 |  |  |  |  |
| Indiana | 63 |  |  |  |  |  | Illinois | 79 |  |  |  |  |
| North Carolina | 63 |  |  |  |  |  | Alabama | 80 |  |  |  |  |
| Illinois | 64 |  |  |  |  |  | North Carolina | 80 |  |  |  |  |
| Michigan | 64 |  |  |  |  |  | South Carolina | 80 |  |  |  |  |
| Iowa | 65 |  |  |  |  |  | Nevada | 82 |  |  |  |  |
| Maryland | 67 |  |  |  |  |  | New Jersey | 83 |  |  |  |  |
| West Virginia | 68 |  |  |  |  |  | Maryland | 85 |  |  |  |  |
| Georgia | 69 |  |  |  |  |  | Florida | 86 |  |  |  |  |
| Louisiana | 69 |  |  |  |  |  | Mississippi | 88 |  |  |  |  |
| Alabama | 71 |  |  |  |  |  | New York | 90 |  |  |  |  |
| Mississippi | 77 |  |  |  |  |  | Georgia | 100 |  |  |  |  |

| TERTILE KEY | | |
| --- | --- | --- |
| **Low RSV** | **Moderate RSV** | **High RSV** |
